# Cultural Influences on Complementary Feeding Beliefs amongst New Chinese Immigrant Mothers in England: A Mixed Methods Study

**DOI:** 10.3390/ijerph17155468

**Published:** 2020-07-29

**Authors:** Xiaoning Zhang, Diana Margot Rosenthal, Lorna Benton, Monica Lakhanpaul

**Affiliations:** 1 School of Nursing, Xuzhou Medical University, 209 Tongshan Road, Xuzhou 221004, China; 2 School of Nursing, Capital Medical University, 10 YouAnMen Xitoutiao, Beijing 100069, China; 3 Population, Policy and Practice Research and Teaching Department, UCL Great Ormond Street Institute of Child Health, 30 Guilford Street, London WC1N 1EH, UK; diana.rosenthal@ucl.ac.uk (D.M.R.); lorna.benton.09@ucl.ac.uk (L.B.); m.lakhanpaul@ucl.ac.uk (M.L.); 4 Collaborative Centre for Inclusion Health, UCL Institute of Epidemiology and Health Care, University College London, London WC1E 7HB, UK

**Keywords:** complementary feeding, feeding beliefs, cultural influences, Chinese immigrant

## Abstract

Adequate nutrition during infancy and early childhood is essential for ensuring the growth, health, and development of children so that they can reach their full potential. There is a current void of data on infant and young child feeding practices (IYCF) in ethnic minority communities in the UK; specifically, it is difficult to find accurate Chinese IYCF data in the UK because survey data often includes Chinese in the category of ‘Chinese or other ethnic group’, further contributing to health inequalities. This mixed methods study aimed to explore the cultural influences on IYCF beliefs among new Chinese immigrant mothers. A total of 31 mothers of infants aged 6–23 months were recruited from informal community organizations. All 31 mothers were born in Mainland China, the mean length of their stay after immigrating to the UK was 10 years (range = 1–21 years), and their mean age was 29 ± 3.40 years. When using the Infant Feeding Style Questionnaire (IFSQ) to investigate IYCF beliefs, the highest score was obtained for responsive attention, with a value of 4.28 ± 0.92, indicating that parents were very attentive to child hunger and satiety cues; lower scores were obtained for indulgence soothing (1.82 ± 1.01), indulgence coaxing (2.11 ± 1.18), indulgence pampering (1.90 ± 0.95), and pressuring to soothe (1.92 ± 0.86), indicating lesser maternal indulgence and pressuring/controlling beliefs. A sub-sample (*n* = 14) participated in semi-structured interviews in order to understand the balancing sources of information and cultural preferences, the influence of traditional Chinese medicine, and language difficulties in accessing health services. The mothers reported barriers of IYCF beliefs and the introduction of solid foods earlier than the NHS guidelines. This study can promote optimal IYCF in Chinese immigrants and show health services the need to reconcile differences between the perceptions of British and Chinese health beliefs.

## 1. Introduction

The first 1000 days of life represents a critical window of time for the prevention of both under- and over-nutrition [1]. Establishing healthy dietary beliefs and behaviors in early life is an important health strategy for combatting childhood overweight and obesity [2]. Although few innate dietary preferences exist at birth [3], both the National Health Service (NHS) and Scientific Advisory Committee on Nutrition (SACN) advise that exclusive breastfeeding for approximately the first 6 months is optimal; six months is also the recommended age to begin introducing appropriate types and amounts of solid foods [4]. Complementary feeding (CF) is defined as the introduction of solid and semi-solid foods along with breast milk when breast milk alone is no longer sufficient to meet the nutritional requirements of infants [5]. The target range of CF is 6 to 23 months of age, with the purpose of developing infant and young child dietary beliefs and behaviors [6]. During this important period of life, infant and young child feeding practices (IYCF) have been identified as an important, under-prioritized strategy for the early prevention of childhood obesity, micronutrient deficiency, and fostering lifelong health. IYCF is a key determinant not only of the nutritional intake of children, but also feeding behaviors. Therefore, it has an influence on not only growth, but also development, which is influenced by a number of cultural and social factors, including the feeding beliefs of mothers. Such factors led to the development of the Infant Feeding Style Questionnaire (IFSQ) due to the need for a valid and reliable measure of parent feeding practices in infancy and early childhood. This questionnaire identified five feeding styles [7] and was used in the present study. These five feeding styles were estimated with internal reliability measures demonstrating several sub-constructs, such as responsive to satiety cues, pressuring with cereal, indulgent pampering, and indulgent soothing, which validated this instrument [8].

Cultural diversity is a typical characteristic of many countries, while race and ethnicity are recognised determinants of health inequalities in urban populations [9]. To address health inequalities, it is important to consider how socio-cultural differences influence minority populations [10,11]. According to the 2011 Office of National Statistics Census, amongst the 56 million people living in England and Wales, 7.5 million (13%) were born outside of the UK; 300,000 (4%) Chinese immigrants made up the total population [12]. More specifically, in 2017, London was the region with the largest number of migrants—3.4 million foreign-born—and China was in the top three nationalities of non-EU citizens receiving UK study visas [13]. In addition, in 2011, there were 724,000 live births in England and Wales (22% increase since 2001) and this growth has been accompanied by a large number of births to non-UK-born women—from 16% to 26% (*n* = 185,000) of the total [14]. As the numbers of Chinese immigrants are increasing rapidly, it is important that the public health needs of this community are addressed. It will also be necessary to recognize the cultural and ethnic diversity within the Chinese community to ensure that interventions and advice are appropriately tailored. Chinese populations are often referred to as a homogenous group. In fact, there are a variety of nationalities, cultural groups, and ethnicities that fall under the label ‘Chinese’ and a lack of clarity surrounding the label, which make this group an exemplar of the complexities of ethnicity and culture and the need to challenge assumptions and stereotypes [15]. A 2010 survey included Chinese in the category of ‘Chinese or other ethnic group’, which is common practice in national survey data [4,16]. In this study, ‘Chinese’ was defined distinctively by self-identification as Chinese from a range of countries or regions, e.g., China, Taiwan, Hong Kong, Malaysia, Vietnam, etc. However, there is a lack of research addressing IYCF in Chinese children, creating a need to explore the traditional cultural and health beliefs of their mothers and associated behaviors, in order to better understand how to optimize their future health and wellbeing.

Cultural and individual characteristics, as well as knowledge, beliefs, and attitudes, have important influences on the perceived behavior of mothers of different ethnicities, cultural dietary preferences, and eating beliefs that shape dietary choices. There is evidence regarding cultural influences on IYCF of Chinese immigrant mothers in Australia, which identified culturally-sensitive guidance of IYCF as a means of promoting and increasing exclusive breastfeeding and maternal confidence, and of mitigating contradictory advice given by Chinese grandparents versus health professionals [17]. IYCF has been recognised to be influenced by culture-specific beliefs [18]; Chinese immigrants’ feeding practices are recognised as quite complex and have been identified in other ethnic groups [19], and the prevalence of obesity in Chinese-American children is lower than the prevalence in other ethnic groups [20]. However, there is a lack of data on the existence of cultural influences on IYCF in minorities in the UK, as well as health policies. Despite this, CF has been identified as an important and under-prioritized strategy for preventing childhood obesity and fostering lifelong health in the UK, specifically in the greater London area, which has the highest concentration of immigrants [13]. The absence of mixed quantitative and qualitative data makes it difficult to extrapolate the impact of culturally-appropriate IYCF across diverse minorities living in the UK. Interventions for addressing feeding practices need to be culturally adapted to different populations given the prominent population of Chinese immigrants in the UK and the evidence for ethnic inequalities, with minorities exhibiting worse health outcomes compared to the white British population [21,22].

The aims of this mixed methods study were (1) to explore how cultural influences affect IYCF beliefs amongst new Chinese immigrant mothers in the UK, and (2) to identify perceived barriers and facilitators to the optimal IYCF of Chinese immigrant mothers and understand these barriers. In turn, the study intended to improve and enrich this research area in order to provide evidence for eventually designing and co-developing a tailored intervention program to help optimize IYCF in the first 1000 days. The theoretical framework of this study was underpinned by the socio-ecological model, which considers that any health behavior has many levels of influence and interplay between several factors, with a focus on the social and/or community environment [23,24]. This study is an extension of the Nurture Early for Optimal Nutrition (NEON) project, which aims to support optimal IYCF in ethnic minority families living in the UK, since feeding practices, food practices, and behaviors during early experiences can carry over into later childhood and adulthood, in an effort to prevent the development of cardiovascular diseases and co-morbidities [25,26,27].

## 2. Methods

### 2.1. Study Design

This cross-sectional mixed methods study used surveys and semi-structured interviews to explore cultural influences on IYCF beliefs among Chinese immigrant mothers using the IFSQ. Qualitative research was used because it allows an open-ended approach with changes of direction and new insights; specifically, the semi-structured interviews allowed for a further in-depth analysis on Chinese mothers’ views of cultural influences on IYCF beliefs [28]. Triangulation was employed to integrate corroboration between quantitative and qualitative data in order to gain a more complete picture [29].

#### 2.1.1. Recruitment

Chinese immigrants have been stereotyped as a ‘hard to reach’ minority group owing to their geographical distribution and language barriers [30], although this study defined the population as marginalized. Purposive sampling [31] is a recognized way of recruiting participants from communities that researchers may otherwise find more challenging to access; this method was used to recruit Chinese mothers across central London—a geographical area where Chinese communities are concentrated—between June 2017 and March 2018; 33% of the Chinese population previously resided in London [16]. This study identified several Chinese community organizations by Google and references living in London to contact: 2/11 agreed to participate as recruitment sites—two Chinese churches (*n* = 21). The remaining participants were recruited through social media apps (mother groups on WeChat, *n* = 10) through purposive sampling in order to further enhance recruitment. Participants were then provided with a summary of the study in either English or Mandarin. Interested participants contacted the research team in person while on site at a community event, by phone, or by WeChat voice; they were then screened for eligibility.

At a mutually agreed convenient location, time, and date, the survey and/or interview was conducted. A private area in a Chinese church was the most common location chosen by participants. Of the total sample (*n* = 31) that participated in the IFSQ, a sub-sample of 14 participants agreed to participate in an interview. In terms of the schedule, some participants attended Church activities irregularly from relatively distant places, and they could not ensure the time that they came to the church; therefore, if convenient and they agreed, participants were offered the option to complete the questionnaires by Google Sheets, and the interview by WeChat voice or telephone. As a token of thanks for completing the IFSQ survey and semi-structured interviews, participants received remuneration through gift cards of £10 and £5 for completing the survey questionnaire and interview, respectively.

The following inclusion criteria were adopted: (a) The mother was of Chinese origin, born outside the UK, migrated to the UK, self-identified as an immigrant from Mainland China, and has been settled in the UK for at least 1 year, and (b) the mother had at least one child aged between 6 and 24 months, and full term. Exclusion criteria included (a) being of mixed Chinese ethnicity and (b) having a child with medical complications. Two researchers conducted participant selection, and the research team resolved disagreements or clarifications over inclusion and exclusion; for example, the original inclusion criteria were broader in order to include women who self-identified as an immigrant from Hong Kong, Vietnam, Singapore, or Malaysia, but they did not participate in the study, which was a limitation of the purposive sampling and eventually only left the participant pool with a selection of Mainland Chinese mothers.

#### 2.1.2. Data Management and Ethical Approval

A researcher (X.N.Z.) who was fluent in English and Mandarin collected data to ensure the validity of this cross-sectional study with cross-cultural application [32]. First, X.N.Z. gave potential Chinese mothers an electronic or verbal study summary and invited them to take part. If they agreed, participants were provided with a written information sheet including the research purpose, expected outcomes, procedures, benefits, risks, and their rights not to take part in either English or Mandarin. All written consent was obtained prior to conducting this study. If participants were recruited by social media, all documents were posted to them to sign and return. This study identified participant literacy through conversation; literate participants were given information sheets including contact details and invited to discuss any issues with the researchers. Oral informed consent procedures were followed for illiterate participants. All data were anonymized and processed according to the British Educational Research Association [33] with regards to data protection and storage. The project was approved for research ethics by the University College London Research Ethics Committee (Ethics project ID number: 10271001).

Human participant approval was obtained from University College London. Participants provided signed consent. Informed written consent for publication was obtained from all participants.

#### 2.1.3. Measure

Before the survey questionnaire and interview, basic demographic information was collected, including age, marital status, place of birth, employment status, education level, length of stay in the UK, and number of children. The IFSQ was conducted via printed paper questionnaires and Google Sheets, which was informed by the suggestion of Costanzo and Woody that parents adopt feeding domain-specific parenting styles [34,35], based on concerns and constraints. The IFSQ included items that assessed maternal IYCF beliefs through the domains of five feeding practices: Laissez-faire (diet quality and attention feeding); pressuring/controlling (finish, cereal, and soothing feeding); restrictive/controlling; responsive (amount and diet quality feeding); and indulgence (permissive, coaxing, soothing,, and pampering feeding) (Table 1). Probed beliefs were coded on a 5-point Likert scale, with 5 = agree, 4 = slightly agree, 3 = neutral, 2 = slightly disagree, and 1 = disagree. Each domain contained multiple subscales for a total of 13 subscales with 48 items [8]. The full survey questionnaire is provided in the Supplementary Materials.

The variables of the IFSQ were validated measures of IYCF, which have been used in previous studies [36,37]. The H coefficient was ≥0.80 for all cases, and the values of three sub-constructs (restrictive amounts, pressuring to finish, and with cereal) were ≥0.75 [8]. In a USA sample, the internal consistency of the subscales under the indulgence, pressuring, restrictive diet quality, and laissez-faire diet quality were ≥0.70, while attention and responsive satiety were ≥0.60 [38]. IFSQ has been reliably measured in Chinese populations [39] through the H coefficient and Cronbach’s alpha = 0.910; each subscale of the Cronbach’s alpha was >0.898.

#### 2.1.4. Interview

Semi-structured interviews [40] were conducted by a bilingual researcher with proficiency in Mandarin (X.N.Z.), which built trust with all of the participants and encouraged them to express their thoughts freely. All interviews were audio recorded, allowing mothers to choose their preferred language—English or Mandarin. All of the mothers expressed their preference for Mandarin, since they stated that they could better express any words in Mandarin than in English. Fourteen interviews were conducted: Nine in a private area in Chinese churches and five through a social media app (by video) or telephone. Interviews ranged from 30 to 70 min in length. Open-ended questions with prompts generated discussions on how culture influences IYCF beliefs, which were developed by a literature review on IYCF in Chinese immigrant mothers and our previous studies. A semi-structured interview guide was first piloted with two mothers to make sure that all of the questions were clear and elicited discussions on the intended themes of IYCF beliefs and cultural influences; no changes were made to the guide based on the pilot, since the questions were clear and facilitated discussions with participants as intended. Interview questions included the following: (1) How do you prepare foods for different meal times with your child? (2) What kind of food do you prefer to feed your children? (3) How do you make the decision to stop feeding your children (portion sizes)? (4) How do you think the food culture is different between the British and Chinese? and (5) How do the different food cultures influence the way in which you feed your children? All interview questions can be found in the Supplementary Materials and were drawn from the NEON study—a study using the Participatory Learning and Action cycle to optimize IYCF of Bangladeshi origin in Tower Hamlets, UK. The current study was built on findings obtained from the NEON study [25,26,27].

#### 2.1.5. Analysis

**Quantitative Analysis.** Descriptive quantitative data were entered into Stata 15 (Stata Corporation, College Station, TX) for Windows, and double-checked for data entry errors. Missing values were replaced by mean values. Infant and young child feeding descriptive variables, such as socio-demographics and IYCF, were analysed for descriptive statistics: mean + standard deviation (SD) and frequency (percent, %).

**Qualitative Analysis.** All interviews were transcribed verbatim from Mandarin by the bilingual researcher (X.N.Z.); she translated all transcriptions into English and back-translated, which were validated by two researchers. Any personal details in the interviews were de-identified. The bilingual translators discussed the discrepancies between the original and then back-translated the Chinese version until both reached a consensus regarding the equivalence of language and culture.

Initial coding, categorizing, and storage of quotes were conducted using NVivo11 software (QSR International). Thematic analysis was employed, the process was guided, and unique and common patterns were identified to extend through the whole interviews [41]. Domains were established from the semi-structured interviews: Codes were generated from the data in an open fashion with no pre-set coding, i.e., a feeding bowl (for which mothers estimated the amount of food of each meal for every child) [42]. These were systematically reviewed in a reflexive iterative manner to develop themes until there were no new findings [43], which allowed new themes and subthemes to emerge inductively. Two researchers compared similar and congruent themes to establish data reliability; they resolved any differences in coding and developing themes and subthemes by reviewing the transcripts again and further discussion, until a consensus was reached.

**Triangulation Analysis.** This study collected and analyzed the qualitative and quantitative data exclusively; the results were then compared and contrasted. Quantitative findings were compared to the themes and quotes generated from qualitative findings. During the analysis and interpretation phases, contention and convergence areas between the qualitative and quantitative data were dissected. A protocol of triangulation was developed in order to (1) produce a convergence coding matrix to display findings emerging from each component of quantitative and qualitative data; (2) consider agreement, partial agreement, silence (findings arising from one data set and not another), or dissonance between findings from different components of quantitative and qualitative data; and (3) list the findings from each component of quantitative and qualitative data, and consider where the findings from each component agree (convergence), offer complementary information on the same issue (complementarity), or contradict each other (discrepancy or dissonance) [29].

## 3. Results

### 3.1. Participant Characteristics

A total of 31 of 39 Chinese immigrant mothers who were approached by the researchers agreed to complete the IFSQ via printed paper questionnaires (*n* = 20) or Google Sheets (*n* = 11). A sub-sample of 14 mothers were interviewed in Chinese churches (*n* = 7) and over the telephone or a WeChat voice call (*n* = 7). Table 2 presents the descriptive characteristics of the sample population.

### 3.2. Quantitative Findings

Table 3 presents the mean IFSQ scores for mothers’ IYCF beliefs. It was found that 90.4% of mothers agreed or slightly agreed that it was important to help or encourage a toddler to eat, while 3.2% of mothers agreed or slightly agreed that toddlers should be allowed to drink sugary drinks/soda to keep them happy or from crying.

### 3.3. The Triangulation of Quantitative and Qualitative Findings

#### 3.3.1. Responsive Belief with Cultural Influences

The responsive feeding style reflected whether the mothers were attentive to the satiety cues and monitored the diet quality of their infants. The mean score of responsive satiety was 3.76 ± 1.15. In total, 67.7% of mothers agreed or slightly agreed that a child knew when she/he was full and 74.1% of mothers agreed or slightly agreed that a child knew to eat when hungry, which was consistent with the interviews. Some mothers believed that their children could feel when they were hungry or full.

*“All of my children understand what is full or hungry. If I feed my children too much food, they would not open their mouths.”*

(id 15: 4 children)

Mothers scored the highest on responsive attention (Table 3). In total, 90.4% of mothers stated that it was important to help or encourage a toddler to eat, which was convergent with the interviews. Most of the mothers encouraged their children to eat a traditional Chinese diet: They stated that the Chinese diet contained more nutrients, and made their children become a ‘chubby baby’, which typically included pork ribs, chicken, or fish with soup.

*“When I fed her new food, she did not like, I fed her the same food repeatedly, and one day she ate a bit, finally, she ate up.”*

(id 7: 4 children)

#### 3.3.2. Restrictive Belief with Cultural Influences

Restrictive reflected the amount and diet quality in which the parents limited the infants’ healthy foods and quantity of food consumed. Of the total sample, 58.1% of mothers agreed or slightly agreed that parents should decide how much their infants should eat, which was partly dissonant with the interviews. Most of the mothers stated that they did not ‘restrict’ their children’s food; however, they believed that the dietary restrictions helped improve their children’s health, and hence highlighted the quality and quantity of food. Most of the mothers fed their children lots of fruits and vegetables; they stated that fruits and vegetables were healthier foods.

The mean score of restrictive diet quality was 3.35 ± 1.18; 54.9% of mothers agreed or slightly agreed that a toddler should only eat healthy food, whilst 58.1% of mothers agreed or slightly agreed that the infant should not eat fast food, which was consistent with interviews. Some mothers believed that low-fat and light foods were healthier choices; they prevented their children from eating fried foods and processed sweets.

*“I only give my children light foods, such as vegetables. I rarely feed them fried foods. I have to cook food by Chinese healthy ways, my children have to eat light food.”*

(id 14: 3 children)

*“I pay special attention on my children’s food, I hope all kinds of food they eat are organic.”*

(id 16: 2 children)

#### 3.3.3. Pressuring Belief with Cultural Influences

Pressuring meant that the parents were concerned with increasing the amount of food their infants consumed and used food to soothe their infants. The mean score of pressuring to finish was 3.58 ± 0.86; 51.6% of mothers agreed or slightly agreed that it was important for a toddler to finish all food on his/her plate, which was convergent with the qualitative data. To understand portion size in this context, the interviews showed that most of the mothers prepared a feeding bowl and might be influenced by extended family.

*“We have a feeding bowl for children respectively, according to the amount for each meal, normally the amount of food my children eat is a bowl.”*

(id 16: 2 children)

*“My mother in law prepares food in a feeding bowl; my children have to eat up all the food in the bowl.”*

(id 27: 3 children)

The mean score of pressuring with cereal was 2.77 ± 0.70; 48.4% of mothers agreed or slightly agreed that an infant needed more than formula or breast milk to be full within 6 months, which was convergent with the interviews. Some mothers introduced solid foods to their children when they were between 4 and 6 months of age, which was earlier than the time recommended by the NHS, although they stated that they understood the differences between Chinese and British feeding practices.

*“My children start solid foods from vegetables and baby rice; they only start baby rice in China, but we eat vegetables, starting with carrots in the UK.”*

(id 3: 4 children)

Some mothers believed that the age of introducing solid foods should be flexible—they estimated that their children were ready to be fed solid foods within 6 months based on existing cognition and solid foods should be first introduced by children showing interest in solid foods.

*“Some children are more advanced, they already showed interest in foods at 5 months of age. When adults ate foods, they wanted to grab our foods, so I tried to feed them solid foods, such as fluffy banana. 6 months should not be the boundary.”*

(id 11: 3 children)

*“In fact, they can start [complementary] feeding at 5 months of age, my first child started solid foods at 4 months of age, the time of complementary feeding should be flexible, 6 months is too strict.”*

(id 7: 4 children)

The mean score of pressuring to soothe (*soothing*) was 1.92 ± 0.86; 12.9% of mothers agreed or slightly agreed that the best way to make infants stop crying is to feed, which was convergent with the interviews. No mothers made this statement during the interview.

#### 3.3.4. Laissez-Faire Belief with Cultural Influences

Laissez-faire indicated that the parents do not limit the infant diet quantity or quality and show little interaction with the infant during contemporary feeding practices. The mean score of laissez-faire attention was 2.63 ± 1.15; 67.8% of mothers disagreed or slightly disagreed that a toddler could walk around as long as they were eating food, which was convergent with the qualitative data. Few mothers stated that they chased their children to eat; one mother highlighted an eating ritual in which her child had to sit in a chair near the table during meal times, and explained how she would not feed her children when they were on the floor.

The mean score of laissez-faire diet quality was 2.17 ± 1.12. Only 16.2% of mothers agreed or slightly agreed that a toddler could eat whatever she/he wanted for snacks, which was partly convergent with the interviews. Most of the mothers stated that they gave healthy snacks to their children between meals, such as fruits, yoghurt, etc.

#### 3.3.5. Indulgence Belief with Cultural Influences

Indulgence reflected that the parents do not limit the quantity or quality of food consumed. The overall mean score of indulgence was the lowest among the IFSQ categories (Table 3). In total, 12.9% and 9.7% of the mothers agreed or slightly agreed, respectively, that toddlers should be allowed to drink sugary drinks/soda if they want or made sure they got enough. Only 3.2% of the mothers agreed or slightly agreed that toddlers should be allowed to drink sugary drinks/soda to keep them happy or from crying. The quantitative results were partly dissonant with the interviews; some mothers stated that they indulged their children in other ways.

*“Milk time may be flexible, sometimes my boy only drinks breast milk before sleeping. Usually my nipples are in his mouth overnight, because he feels safe.”*

(id 8: 3 children)

### 3.4. The Barriers and Facilitators of Cultural Influences

#### 3.4.1. Balancing Sources of Information and Cultural Preferences

All mothers stated that they received feeding practice support and assistance from health professionals (e.g., a midwife, health visitor, children’s center, etc.), the NHS website, publications, peer support groups, and friends and family; most of them balanced and compared different information sources of IYCF, especially Chinese and Western health beliefs. Only three mothers strictly followed the advice of health professionals, which was dissonant with several Chinese health beliefs, i.e., one mother breastfed until her child was two years old. For some mothers who had lived in the UK for a longer time, they still relied on Chinese information sources: one of the mothers only obtained IYCF information from Chinese social media and relatives living in China. Through Chinese churches, one of the mothers only received information from an old Chinese version feeding book containing outdated feeding practice advice. Some mothers worried that their children could not eat Chinese food in school, so they prepared British food to adjust to school meals at home.

*“Daily information from church friends is more practical, they give me lots of details. Midwives give same information to my four children, for example no water during breastfeeding, though which is actually in conflict with Chinese methods. But I think they are midwives, they raise children like this in the UK, and they emphasize it, definitely it is no harms to my children, so I follow their advice.”*

(id 15: 4 children)

Most of the mothers preferred to seek peer support when the information sources provided conflicted with traditional Chinese feeding beliefs or when the information was contradictory. Instead, they accessed social media resources, such as WeChat mother support groups, which were very popular in this study, although one of the mothers stated that she got absurd advice.

*“We have online mums’ groups, discussing feeding information, if we couldn’t agree with, I would communicate with health visitor.”*

(id 25: 2 children)

#### 3.4.2. Cultural Influences and Feeding Conflicts

Some mothers stated that from their perspective, the body composition of Chinese and British people was different, and therefore exhibited perceptions of feeding for body composition. Some mothers believed that living in the UK meant that they should follow the British feeding beliefs and customs and abandon those of China. One mother believed that the Chinese body composition or medicine should be the same as the British, stating that she was British but just born in China.

*“We are British now, we have to obey British feeding and listen to the British professionals, I do not think British and Chinese have different body composition.”*

(id 15: 4 children)

*“Though I have been the UK for a long time, I still think Chinese diet is relatively healthy, because the body composition of my children is different with British, I must cook Chinese foods, rarely feed them junk foods.”*

(id 16: 2 children)

Most of the mothers always applied Chinese dietary and traditional culture; they disagreed with health professionals about feeding water during feeding times and exclusive breast milk for the first 6 months; this represents only one example of disagreement between Chinese and British IYCF.

*“My three children have three different feeding styles, first child fed by my mother-in-law, complementary foods were Chinese porridge with egg yolk. Second child fed by my own mum, complementary foods were Chinese thin soup. I fed third child by myself following midwives, for example cutting avocado into small pieces, ate by herself.”*

(id 27: 3 children)

#### 3.4.3. The Influence of Traditional Chinese Health and Feeding Beliefs

Some mothers explained that several Chinese health beliefs and cultural influences are related to their IYCF. Most of the mothers preferred hot foods, water, milk, and tea, and avoided cold foods or drinks; they applied traditional Chinese cooking methods, including foods that were not of Chinese origin, e.g., pasta, in the same style as Chinese soup noodles.

Some mothers followed the traditional Chinese health beliefs of “*Shanghuo*”*,* which referred to “redness, swelling, fever, and pain” [44]; they assessed their children’s digestion and absorption according to the appearance of their feces. When their children had difficulty with passing stools, mothers believed that this was a symptom of *Shanghuo*, and one mother stated that eating apricots was related to constipation.

Some mothers applied traditional Chinese dietary “*Tufang”*, which was mainly disseminated by word of mouth: One mother used pear boiled with water or oranges steamed with salt to treat her children’s feeding problems, while another mother tried ginger boiled with water to prevent stomach flu. Only one mother sought treatment from traditional Chinese medicine practitioners in London.

*“I use traditional Chinese medicine, all generations of my hometown drink one kind of herb, I can find them in London’s parks, soaking with water to drink directly.”*

(id 20: 3 children)

#### 3.4.4. Language and Cultural Barriers in the Access of Health Services

Some mothers stated that they experienced communication difficulties leading to misunderstandings of recommendations and treatment by health professionals, especially those who had different health beliefs from those within the Chinese community; the consequence was that they reduced their use of health services:

*“If my babies got diarrhea for a few days, we might go to the emergency, but usually doctors don’t give any suggestions, they only give some salty water. My first baby had a fever, doctors only gave Febrifuge, taking off all the clothes, and therefore I didn’t take my second and third babies to the hospital. We prepare Antipyretic at home, if they got fever, toothache and tickle, I fed them medicine directly.”*

(id 8: 3 children)

Regardless of the duration that they had been living in the UK, all mothers preferred to be interviewed in Mandarin; most of them communicated IYCF information in Mandarin. Three mothers reported their English barriers.

*“My first two children, they needed to inject vaccines within one year old, but because my English was not good and some information, I didn’t see either, as a result, both missed vaccinations, no friends at that time told me and probably they thought I should understand, but I didn’t know, so I missed their vaccinations.”*

(id 8: 3 children)

A distinction shown by mothers was that when they went to the hospital, they might have considered this as ‘to see my disease/body,’ rather than ‘to see a doctor.’ When a mother took her child to see a general practitioner, any advice that did not include the use of medicine, which may not have been in line with Chinese health beliefs, or may not have been acceptable, especially when the expected health outcome did not appear, created more discordance and doubt. However, when doctors showed an awareness and knowledge of different cultural contexts, mothers were more willing to trust and engage in those services:

“Basically, I can understand all my GP ’s words, he is very kind and has more patience to explain. Sometimes if I don’t understand, he would draw a picture for me. He understands Chinese culture because he knows what kind of foods Chinese like, the information he gives is useful for Chinese, health service relates to doctor’s suggestions in different people and races. My GP has extensive knowledge according Chinese cultural context”

(id 20: 3 children)

## 4. Discussion

Cultural influences and individual health beliefs and behaviors played important roles in IYCF. This study integrated qualitative and quantitative data, allowing for the identification of convergent and divergent findings, which demonstrated a deeper understanding of the cultural influences on IYCF among Chinese immigrants. To the best of our knowledge, this was the first mixed methods study to explore the cultural influences on IYCF beliefs among new Chinese immigrant mothers in the UK. Chinese immigrant mothers reported high responsive attention and satiety levels and low indulgence feeding levels; they engaged in advocating healthy dietary beliefs and benefited from the balancing of information sources, although there were cultural conflicts. Chinese mothers reported barriers of first introducing solid foods earlier and language difficulties when accessing health services. In an effort to increase the understanding of the culturally-appropriate and optimal IYCF, the objective was also to learn what a tailored intervention program might look like across minority groups in the UK.

### 4.1. Summary of Findings

The findings of this exploratory study showed that the beliefs of laissez-faire, pressuring, restrictive, and responsive were more frequently described among Chinese immigrants than a white middle-class population [36]. Restricting unhealthy foods by preventing access to and/or limiting the amount consumed was a general strategy for improving infants’ diet or reducing the risk of weight gain. The consistent findings of the surveys and interviews showed that new Chinese immigrant mothers believed that the dietary restrictions helped to improve infant and young child health and wellbeing, and hence highlighted the importance of the quality and quantity of food [17]. In comparison to a cross-sectional analysis of an ethnically diverse sample in the USA [38], the mean scores of laissez-faire beliefs for attention and diet quality, and pressuring to finish and with cereal were higher than average, while pressuring to soothe was consistent with the sample. The mean scores of Chinese immigrant mothers’ beliefs on restrictive amounts and diet quality were higher than average when compared with a diverse USA sample [38], which indicated that the mothers engaged in highly restrictive feeding practices aimed at distracting the infant or young child from satiety by concentrating on restricted foods, whereas greatly pressuring was believed to boost eating in the absence of satiety [45]. Controlling was often measured by pressuring and restrictive to IYCF [35]. Chinese parents have been described as highly controlling, and even labelled as authoritarian and ingrained in traditional culture [46]. Commanding mothers balancing demand and responsiveness have been associated with a lower BMI in older children [47], and positive IYCF beliefs.

Laissez-faire demonstrated that the mothers were lenient (64.5%) regarding what snack foods they gave their children, although most stated that they gave “healthy snacks.” However, restrictive feeding styles may be associated with a poorer self-regulation of appetite and decreased intake of fruits and vegetables [48], despite being a common strategy to improve children’s diets and reduce the risk of unhealthy weight gain. Fruit and vegetable consumption could be related to obesity prevention [49], but this may result in children showing a later preference for restricted food and increases in its consumption once the restriction is removed, and therefore also increase the risk of obesity [48]. In this study, the majority of the Chinese immigrant mothers described feeding their children lots of fruits and vegetables, but restricted both foods and drinks high in sugar; they stated that fruits and vegetables were healthier foods, which is in alignment with national health advice, preventing early tooth decay. Extremely controlling feeding practices have been observed to have a detrimental influence on the regulation of appetite among infants and young children [50], whilst responsive to satiety has been linked to a reduction of the obesity risk [51]. Indulgence has been associated with a higher BMI and lower nutrient intake in older children [52], which included responsive soothing to the children with abundant demands. In this study, new Chinese immigrant mothers engaged in low indulgence, which was higher than a USA sample [38].

Though the majority of the Chinese mothers fed children by themselves, the approaches used and the way in which the mothers fed their children were culturally influenced by extended family [17]. Chinese immigrant mothers explained some differences in their Chinese health beliefs and cultural influences: The majority of them preferred the traditional Chinese diet [17]. Some Chinese immigrant mothers strictly followed the advice of health professionals; these mothers believed British health beliefs and abandoned Chinese health beliefs, which was inconsistent with an Australian qualitative study on factors influencing Chinese immigrant mothers’ early feeding choices and perceptions of infant growth that was conducted to identify barriers and facilitators to the appropriation of optimal IYCF [17]. However, the mothers did engage in pressuring their children to eat custom Chinese delicacies, including soup mixed with pork, chicken, or fish, which are all notably high in sodium, and the NHS and SACN strictly advise against foods with a high salt content, in order to prevent kidney problems. During the interviews, there was no mention at all of vitamins for both the mother and baby; national guidelines recommend that is vital for a baby to receive Vitamins A, C, and D every day for optimal growth and development. In addition, more than half of the mothers believed that their toddlers should finish all of the food on their plate, which is not nationally recommended; they pressured their toddlers to eat a certain quantity through the use of a food bowl. In contrast, responsive feeding is both recommended and mutually important for mothers and infants for building a healthy and loving close relationship [5,53]. The NHS and policymakers need to focus on engaging with this community pertaining to the parents’ and caregivers’ attitudes, beliefs, and behavior about food and feeding, in order to ensure the safety and optimal nutrition of these children for growth and development.

One study reported that Chinese immigrant mothers were more likely to introduce solids earlier in the USA [54]. In this study, most of the mothers introduced solid foods between 4 and 6 months, which was influenced by Chinese culture and an early interest in food; this was in agreement with previous studies [54,55,56]. It was shown to be common to introduce solid foods and other non-milk liquids to infants younger than three to four months in some parts of China, which was likely to be culturally linked to this practice [57]. In this study, the majority of the Chinese mothers introduced solid foods earlier than advised by the NHS guidelines. One important factor influenced by Chinese health beliefs was that the mothers estimated that their children were ready to be fed solid foods within 6 months of age based on Chinese feeding practices. However, the NHS and UNICEF UK strictly recommend that solid foods are not introduced before the infant is able (1) to stay in a sitting position and hold their head steady; (2) coordinate their eyes, hands, and mouth, which will allow them to look at food, pick it up, and put it in their mouth by themselves with ease; and (3) swallow food without spitting it out. However, often, parents may misinterpret that their baby is ready for solid foods by signs such as chewing with their fists, waking up in the night more than usual, and wanting extra milk feeds [5,58]. The early introduction of solid foods is dangerous alongside the underutilization of health services or discordance with the advice given by health visitors, because the mothers miss out on key health and safety guidance, such as food preparation, what foods to avoid (i.e., detecting allergies to eggs, nuts, fish, and wheat), and how to detect whether their baby is choking rather than gagging. The NHS and SACN recommend that foods containing allergens not be introduced prior to 6 months of age, and when they are introduced, this needs to be done one at a time, in order to spot any potential reaction. During the interviews, the mothers did not make any reference to food allergens, or if they knew the signs indicating that their baby was choking or if they had experienced it. Doub et al. (2015) found that the early introduction of solid foods was more prevalent among mothers who were younger, less educated, and heavier pre pregnancy, and who breastfed for shorter durations and reported a lower responsiveness to their infants’ hunger and satiety cues and that their infants needed more than milk at <6 months [36]. Rogers and Blissett (2018) found that mothers with higher scores of laissez-faire feeding behaviors did not keep track of how much milk their infant drank at 3 months, which was associated with the earlier introduction of solid foods [59]. Therefore, in the development of an intervention, it might be worthwhile to consider a focus on the timing of the introduction of solid foods and safety and hygiene guidance for this community, as well as co-production with the mothers, in order to address their needs from both sides.

### 4.2. Implications

The results highlighted the therapeutic and comforting aspects of foods, and how diet and traditional Chinese medicine are inseparable in traditional culture health beliefs, with long-established beliefs that a particular diet has health-promoting or -damaging properties [60]; this is not only related to nutrients, but also plays an important role in preventing diseases and maintaining health [61] and diets [62].

Findings from this mixed study demonstrated that Chinese immigrant mothers tended to encourage healthy dietary beliefs and identify positive cultural ones associated with IYCF. It became apparent that they were confused by contradictory and conflicting sources of IYCF information [63]. When the information provided by health professionals was not in line with traditional Chinese health beliefs, they benefited from some form of peer support. Building official online supportive groups might assist Chinese immigrant mothers in achieving optimal IYCF and help health professionals to understand their feeding beliefs and behaviors. In this study, Chinese mothers balanced CF knowledge from different sources of information; they advocated light and healthy dietary beliefs—an explanation for this was that some of the mothers followed the approach of ‘*yangsheng*’—and the use of self-healing and nurturing the body to cultivate personal health and longevity [64].

Chinese immigrants have consistently underused health care facilities and services compared to other ethnic minorities and white populations in the UK [65]. In this study, barriers to the uptake of services included linguistic misunderstanding and cultural influences; such beliefs are that English doctors will not understand the feeling of Chinese women, and such women are reportedly scared of seeing or speaking to the doctor or believe that the doctor did not listen to them [66]. Cultural differences acted as an important barrier to health service uptake among the Chinese population due to individuals’ concerns about their ability to communicate with health professionals [67,68]. Traditional culture and health beliefs can produce misconceptions around IYCF beliefs [69]. Familiarity with Mandarin and understanding of Chinese traditional culture would improve health professionals’ access to new Chinese immigrant groups and help Chinese infants and young children to obtain optimal IYCF. Language difficulties and the avoidance of health services due to poor interactions can have long-term childhood health outcomes, including the delay of social skills, speech and language, visual or motor functioning, and reaching milestones for particular skills at specific ages [70,71]. For many minority groups, there are barriers and facilitators that influence the uptake of health services and optimal IYCF, including the income level, lack of knowledge, and incorrect advice [27]. The first 1000 days of life is a critical window of time for the prevention of both under- and over-nutrition [1]. Therefore, it is vital to understand what cultural and language barriers exist for this population.

Some of the Chinese mothers had not yet integrated into British society and were also equally far away from the developing Chinese society. Despite living in England for a long time, researchers readily observed isolation, as well as a lack of progress in their routine, daily lives in British society, and updated feeding practices in terms of Chinese feeding practices, which could have been influenced by the fact that the majority of the Chinese immigrant mothers were housewives. Intrinsic features are that Chinese immigrant mothers maintained a Chinese living style in England and habitually still ate Chinese foods and maintained Chinese friendships, language, beliefs, rules, values, etc. It is not a matter of whether these mothers need to “adopt” British ways or feeding patterns or that there may be deficiencies in their practices, but it is vital that there is a way to monitor whether infants and children are reaching their milestones or receiving the correct amount of vitamins; however, this cannot be done if mothers feel discouraged from seeking health advice and using health care services. Through better community engagement and the use of participatory learning action approaches or peer support groups, health professionals can be better educated so that they are aware of Chinese practices and consider the best way to support these families by integrating cultural practices with optimal feeding practices. As the UK continues to be increasingly populated by a diversity of immigrants, culturally competent health services are vital for meeting health care needs and improving health outcomes, patient-provider communication, service delivery, and patient satisfaction.

### 4.3. Strengths and Limitations

The aim of this mixed methods approach was to obtain a deeper understanding of Chinese culture and health beliefs and their influences on IYCF in the UK. A key strength of this study was that we allowed all of the participants to choose their desired and most comfortable language when they were interviewed. The researcher could speak Mandarin fluently and had first-hand materials of Chinese language and traditional Chinese cultural terms and context. This study has filled a gap, as there was once little data on CF in the UK, particularly in Chinese communities. Despite this gap, CF has been identified as an important and under-prioritized strategy for preventing childhood obesity, but also preventing micronutrient deficiency and fostering lifelong health among Chinese populations in the greater London area. This study demonstrated that interventions for addressing feeding practices need to be culturally adapted to different populations living in the UK and more information about the process of cultural influences upon feeding practices is needed. There were barriers and facilitators to IYCF in alignment with the national guidelines in Chinese populations living in the UK, which can result in long-term health outcomes and development delays. The increased prevalence of various adult chronic diseases among ethnic minority populations requires an investigation of the diet quality of infants to determine whether interventions are needed for this demographic, since early intervention can prevent long-term chronic health complications. The findings contributed to the study of why developing or performing healthy and appropriate feeding practices is important, which may allow, in turn, for the design of intervention programs to target their needs.

However, the limitations of language might have resulted in selection bias. The authors attempted to include a broader targeted Chinese group with traditional cultures and health beliefs, with backgrounds that could inform misconceptions around IYCF beliefs. The main limitation of this study was that the samples were not nationally representative of Chinese immigrants, which limited the authors’ ability to compare them with the broader population of Chinese immigrants living in the UK. Participants were recruited through purposive sampling, which produced a selection bias in that all of the participants had Mainland Chinese origins; this limited the conclusions that can be drawn from the study. The sample size was small and there was no comparison group because the lack of tools that assess IYCF designed for minority ethnic groups in high-income countries limits the ability to make relevant comparisons with other studies considering either low-income countries or European and Western ethnicities. Further study is required to explore the issues identified in this study in more minority groups.

## 5. Conclusions

This study showed that new Chinese immigrant mothers need culturally-tailored health policies of optimal IYCF. Challenges arising from the differences between British and Chinese health beliefs and traditional culture, in addition to the differences between home culture and the dominant society, might have influenced new Chinese immigrant mothers in England in terms of how they delivered complementary foods. Promoting optimal IYCF in new Chinese immigrant groups requires health strategies that reconcile the differences between the perceptions of British and Chinese health beliefs and traditional culture. Findings from the mixed methods approach demonstrated that new Chinese immigrant mothers reported positive IYCF beliefs: (1) Engagement in low indulgence and highly responsive to advocating light food and healthy dietary beliefs; (2) the balance of information sources and cultural preferences; and (3) the influence of traditional Chinese medicine. This demonstrated that new Chinese immigrant mothers reported barriers of IYCF beliefs, including introducing solid foods earlier than the NHS guide, language difficulties, and cultural influences on the access of health services.

Optimal IYCF should be re-defined by cultural influences for minorities in the UK. The initial evidence demonstrated that a participatory approach, including peer support which the mothers currently used, would be an ideal strategy because it would enable Chinese immigrants to discuss their options and solutions with health services, rather being told what to do in a top-down manner from someone who does not know their culture at all. The ultimate goal is to achieve culturally-supportive health policies of IYCF; however, studies exploring the views of British and minority health beliefs and traditional culture are warranted to ensure optimal IYCF in minority populations. Further studies specifically investigating dietary diversity through questionnaires are needed, so that health services can understand whether the diets are healthy or there are any deficiencies that they need to support families with, which would benefit from directly co-developing interventions with the community. Health messages need to be tailored and health services need to find a positive way to engage with marginalized communities through sufficient training; IYCF must recognize and be sensitive to cultural practices, but also identify approaches that ensure positive feeding behaviors and dietary diversity during the first 1000 days. This study showed that the culturally-tailored delivery of health information and services was more acceptable and effective in Chinese immigrant groups, and therefore, evidence-based interventions are needed to support optimal and culturally-tailored IYCF in the UK. In the future, we hope to design and co-produce an intervention which links health services and facilitates Asian communities to achieve optimal IYCF.

## Figures and Tables

**Table 1:** Infant Feeding Style Questionnaire (IFSQ) structure and content.

Feeding Style	Sub-Construct	Description
**Laissez-faire**	Diet quality	Parent has no limits regarding food quality or quantity
	Attention	Parent has little or no interaction with child during feeding
**Pressuring**	Finish	Parent controls feeding because of concerns that child is undereating
	Cereal	Parent uses infant cereal to fill child or soothe
	Soothing	Parent feeds child to soothe
**Restrictive**	Amount	Parent limits quantities of all foods
	Diet quality	Parent limits child’s diet to unhealthy foods
**Responsive**	Satiety	Parent is attentive to child’s cues while setting appropriate limits
	Attention	Parent encourages exploration in a positive environment
**Indulgence**	Permissive	Parent does not set limits on the quantity or quality of food consumed
	Coaxing	Parent does not set limits on the quantity or quality of food consumed to ensure child gets enough
	Soothing	Parent does not set limits on the quantity or quality of food consumed to soothe child
	Pampering	Parent does not set limits on the quantity or quality of food consumed to make child happy

**Table 2:** Characteristics of the sample population (*n* = 31).

Characteristic	Mean	SD
Age (years)	29	4.5
Living in the UK (years)	10	7.5
Number of children	3	1
	***n***	**%**
Born in Mainland China	31	100
Married	28	90.3
Housewives	21	67.7
Complete the IFSQ via printed paper questionnaires	20	64.5
**Education**		
No schooling	0	0
Some grade/primary school	1	3.2
Completed grade/primary school	2	6.5
Some high/secondary school	5	16.1
Completed high/secondary school	2	6.5
Some technical, community college	3	9.7
Completed technical, community college	3	9.7
Some university	3	9.7
Completed university	11	35.5
Post graduate degree	1	3.2
Don’t know	0	0
**Socioeconomic status (work)**		
Unskilled	0	0
Skilled work	2	6.5
White collar (office) work	3	9.7
Professional	2	6.5
Not currently working	24	77.3

SD, standard deviation.

**Table 3:** IFSQ in beliefs of five domains scores (*n* = 31).

	IFSQ in Beliefs of Five Domains Scores (Mean ± SD)	Agree (%)	Slightly Agree (%)	Neutral (%)	Slightly Disagree (%)	Disagree (%)
Laissez-Faire	**Attention** (2.63 ± 1.15)					
	*I think it is okay to prop an infant’s bottle*	25.8	12.9	25.8	16.1	19.4
	*It’s okay for a toddler to walk around while eating as long as s/he eats*	16.1	3.2	12.9	25.9	41.9
	**Diet quality** (2.17 ± 1.12)					
	*A toddler should be able to eat whatever s/he wants for snacks*	6.5	9.7	19.3	28.1	36.4
	*A toddler should be able to eat whatever s/he wants when eating out at a restaurant*	6.5	6.5	19.3	35.5	32.2
Pressuring	**Finish** (3.58 ± 0.86)					
	*Important for toddler finish all food on his/her plate*	22.6	29.0	38.7	6.5	3.2
	*Important for infant finish all milk in his/her bottle*	6.5	9.7	35.5	29.0	19.3
	**Cereal** (2.77 ± 0.70)					
	*Cereal in bottle helps infant sleep through the night*	6.5	12.9	22.6	19.3	38.7
	*Putting cereal in bottle good to help infant feel full*	12.9	6.5	25.8	22.6	32.2
	*An infant <6 months needs more than formula or breastmilk to be full*	22.6	25.8	29.0	12.9	9.7
	*An infant <6 months needs more than formula or breastmilk to sleep through the night*	25.8	29.0	22.6	16.1	6.5
	**Soothing** (1.92 ± 0.86)					
	*Best way to make infant stop crying is to feed*	0	12.9	16.1	19.3	51.7
	*Best way to make toddler stop crying is to feed*	0	9.7	19.4	16.1	54.8
	*When infant cries, usually means s/he needs to be fed*	0	16.1	35.5	12.9	35.5
Restrictive	**Amount** (3.45 ± 1.25)					
	*Important parent has rules re: how much toddler eats*	22.6	32.3	28.9	9.7	6.5
	*Important parent decides how much infant should eat*	29.0	29.1	19.3	12.9	9.7
	**Diet Quality** (3.35 ± 1.18)					
	*A toddler should never eat fast food*	19.3	32.4	16.1	16.1	16.1
	*An infant should never eat fast food*	38.8	19.3	6.5	16.1	19.3
	*A toddler should never eat sugary food like cookies*	9.7	22.6	32.2	22.6	12.9
	*A toddler should never eat junk food like chips*	19.3	25.8	25.9	19.3	9.7
	*A toddler should only eat healthy food*	15.6	39.3	19.3	19.3	6.5
Responsive	**Satiety** (3.76 ± 1.15)					
	*Child knows when s/he is full*	32.2	35.5	16.1	9.7	6.5
	*Child knows when hungry, needs to eat*	35.4	38.7	9.7	6.5	9.7
	**Attention** (4.31 ± 0.85)					
	*Important to help or encourage a toddler to eat*	45.2	45.2	3.2	3.2	3.2
Indulgence	**Permissive** (2.23 ± 1.20)					
	*Toddlers should be allowed to watch TV while eating if they want*	3.2	12.9	19.3	19.3	38.9
	*Toddlers should be allowed to eat fast food if they want*	6.5	19.3	12.9	32.3	29.0
	*Toddlers should be allowed to drink sugared drinks/soda if they want*	3.2	9.7	12.9	32.3	41.9
	*Toddlers should be allowed to eat desserts/sweets if they want*	0	29.0	12.9	22.6	35.5
	**Coaxing** (2.11 ± 1.18)					
	*Toddlers should be allowed to watch TV while eating to make sure they get enough*	9.7	25.8	12.9	9.7	41.9
	*Toddlers should be allowed to eat fast food to make sure they get enough*	6.5	6.5	19.4	25.8	41.8
	*Toddlers should be allowed to drink sugared drinks/soda to make sure they get enough*	6.5	3.2	16.1	25.8	48.4
	*Toddlers should be allowed to eat desserts/sweets to make sure they get enough*	6.5	9.7	16.1	29.0	38.8
	**Soothing** (1.82 ± 1.01)					
	*Toddlers should be allowed to watch TV while eating to keep them from crying*	6.5	12.9	16.1	16.1	48.4
	*Toddlers should be allowed to eat fast food to keep them from crying*	3.2	9.7	12.9	29.0	45.2
	*Toddlers should be allowed to drink sugared drinks/soda to keep them from crying*	0	3.2	25.8	25.8	45.2
	*Toddlers should be allowed to eat desserts/sweets to keep them from crying*	0	12.9	22.6	25.8	38.7
	**Pampering** (1.90 ± 0.95)					
	*Toddlers should be allowed to watch TV while eating to keep them happy*	6.5	19.4	16.1	19.4	38.6
	*Toddlers should be allowed to eat fast food to keep them happy*	3.2	12.9	16.1	29.0	38.8
	*Toddlers should be allowed to drink sugared drinks/soda to keep them happy*	0	3.2	12.9	35.5	48.4
	*Toddlers should be allowed to eat desserts/sweets to keep them happy*	0	16.1	12.9	35.5	35.5

SD: Standard deviation.

## Supplementary Materials

The following are available online at https://www.mdpi.com/1660-4601/17/15/5468/s1.
