# Supplementary material for: Cultural Influences on Complementary Feeding Beliefs amongst New Chinese Immigrant Mothers in England: A Mixed Methods Study
Source: Int J Environ Res Public Health. 2020 Jul 29;17(15):5468. doi: 10.3390/ijerph17155468 (PMC7432469; doi:10.3390/ijerph17155468)
Supplement: Supplementary file 1 [file ijerph-17-05468-s001.pdf]

Supplementary Materials

Infant Feeding Styles Questionnaire (IFSQ)- Survey Questions and Quantitative Component

	Feeding Style	Item Description	1	2	3	4	5
**Laissez-Faire**							
Attention		*Behavior items*					
	LF1	When (name of child) has/had a bottle, I prop/propped it up					
	LF2	(Child) watches TV while eating					
	LF3	I watch TV while feeding (child)					
		Belief items					
	LF4	I think it is okay to prop an infant's bottle					
	LF5	It's okay for a toddler to walk around while eating as long as s/he eats					
Diet quality		*Behavior items*					
	LF6	I keep track of what food (child) eats [d]					
	LF7	I keep track of how much food (child) eats [d]					
	LF8	I make sure (child) does not eat sugary food like candy, ice cream, cakes or cookies [C, d]					
	LF9	I make sure (child) does not eat junk food like potato chips, Doritos and cheese puffs[C, d]					
		Belief items					
	LF10	A toddler should be able to eat whatever s/he wants for snacks					
	LF11	A toddler should be able to eat whatever s/he wants when eating out at a restaurant					
**Pressuring**							
Finishing		*Behavior items*					
	PR1	Try to get (child) to finish his/her food					
	PR2	If (child) seems full, encourage to finish anyway					
	PR3	Try to get (child) to finish breastmilk or formula					
	PR4	Try to get (child) to eat even if not hungry					
	PR5	Insist retry new food refused at same meal					
	PR6	Praise after each bite to encourage finish food					
		Belief Items					
	PR7	Important for toddler finish all food on his/her plate					
	PR8	Important for infant finish all milk in his/her bottle					
Cereal		*Behavior items*					
	PR11	Give/gave (child) cereal in the bottle					
		Belief items					
	PR12	Cereal in bottle helps infant sleep thru the night					
	PR13	Putting cereal in bottle good b/c helps infant feel full					
	PR14	An infant <6 mo needs more than formula or breastmilk to be full					
	PR15	An infant <6 mo needs more than formula or breastmilk to sleep through the night					
Soothing		*Behavior items*					
	PR16	When (child) cries, immediately feed him/her					
		Belief items					
	PR17	Best way to make infant stop crying is to feed					
	PR18	Best way to make toddler stop crying is to feed					
	PR19	When infant cries, usually means s/he needs to be fed					
**Restrictive**							
Amount		*Behavior items*					
	RS1	I carefully control how much (child) eats					

	RS2	I am very careful not to feed (child) too much					
		Belief Items					
	RS3	Important parent has rules re: how much toddler eats					
	RS4	Important parent decides how much infant should eat					
Diet Quality		*Behavior items*					
	RS5	I let (child) eat fast food[c]					
	RS6	I let (child) eat junk food[c]					
		Belief items					
	RS7	A toddler should never eat fast food					
	RS8	An infant should never eat fast food					
	RS9	A toddler should never eat sugary food like cookies					
	RS10	A toddler should never eat junk food like chips					
	RS11	A toddler should only eat healthy food					
**Responsive**							
Satiety		*Behavior items*					
	RP1	(Child) lets me know when s/he is full					
	RP2	(Child) lets me knows when s/he is hungry					
	RP3	I let (child) decide how much to eat					
	RP4	I pay attention when (child) seems to be telling me that s/he is full or hungry					
	RP5	I allow (child) to eat when s/he is hungry					
		Belief Items					
	RP6	Child knows when s/he is full					
	RP7	Child knows when hungry, needs to eat					
Attention		*Behavior items*					
	RP8	Talk to (child) to encourage to drink formula/breastmilk					
	RP9	Talk to (child) to encourage him/her to eat					
	RP10	Show (child) how to eat by taking a bite or pretending to[c]					
	RP11	I will retry new foods if they are rejected at first[c]					
		Belief items					
	RP12	Important to help or encourage a toddler to eat					
**Indulgence**[e]							
Permissive		*Behavior items*					
	ID1	Allow child watch TV while eating if s/he wants					
	ID2	Allow child to eat fast food if s/he wants[c]					
	ID3	Allow child to drink sugared drinks/soda if s/he wants[c]					
	ID4	Allow child to eat desserts/sweets if s/he wants[c]					
		Belief Items					
	ID5	Toddlers should be allowed to watch TV while eating if they want					
	ID6	Toddlers should be allowed to eat fast food if they want					
	ID7	Toddlers should be allowed to drink sugared drinks/soda if they want					
	ID8	Toddlers should be allowed to eat desserts/sweets if they want					
Coaxing		*Behavior items*					
	ID9	Allow child watch TV while eating to make sure s/he gets enough					
	ID10	Allow child to eat fast food to make sure s/he gets enough[c]					
	ID11	Allow child to drink sugared drinks/soda to make sure s/he gets enough[c]					
	ID12	Allow child to eat desserts/sweets to make sure s/he gets enough[c]					
		Belief Items					
	ID13	Toddlers should be allowed to watch TV while eating to make sure they get enough					

	ID14	Toddlers should be allowed to eat fast food to make sure they get enough					
	ID15	Toddlers should be allowed to drink sugared drinks/soda to make sure they get enough					
	ID16	Toddlers should be allowed to eat desserts/sweets to make sure they get enough					
Soothing		*Behavior items*					
	ID17	Allow child watch tv while eating to keep him/her from crying					
	ID18	Allow child to eat fast food to keep him/her from crying[C]					
	ID19	Allow child to drink sugared drinks/soda to keep him/her from crying[C]					
	ID20	Allow child to eat desserts/sweets to keep him/her from crying[C]					
		Belief Items					
	ID21	Toddlers should be allowed to watch tv while eating to keep them from crying					
	ID22	Toddlers should be allowed to eat fast food to keep them from crying					
	ID23	Toddlers should be allowed to drink sugared drinks/soda to keep them from crying					
	ID24	Toddlers should be allowed to eat desserts/sweets to keep them from crying					
Pampering		*Behavior items*					
	ID25	Allow child watch tv while eating to keep him/her happy					
	ID26	Allow child to eat fast food to keep him/her happy[C]					
	ID27	Allow child to drink sugared drinks/soda to keep him/her happy[C]					
	ID28	Allow child to eat desserts/sweets to keep him/her happy[C]					
		Belief Items					
	ID29	Toddlers should be allowed to watch tv while eating to keep them happy					
	ID30	Toddlers should be allowed to eat fast food to keep them happy					
	ID31	Toddlers should be allowed to drink sugared drinks/soda to keep them happy					
	ID32	Toddlers should be allowed to eat desserts/sweets to keep them happy					

48 items probe beliefs (code on a 5-point scale: disagree, slightly disagree, neutral, slightly agree, agree) and 57 items probed behaviours (coded on a 5-point scale: never, seldom, half of the time, most of the time, always).

Interview Questions- Qualitative Component

Introduction

1.Please tell me a bit your thoughts about introducing solid foods to your child
a. How do you prepare foods for different meal times with your child?
b. What kind of food do you prefer to feed your children?

Complementary feeding practices

2. Where do you find information about feeding practices?
a. Chinese family, friends or practitioner; British family, friends or NHS health services?
b. What kind of feeding practices information did they give you?
c. Which do you feel are most important?

3. What do you think of when you picture a healthy child?
a. Do you think food at a young age effects health in adulthood?

4. How do you prepare foods for different meal times with your child?

5. What kind of food do you prefer to buy for your child
a. Where from

6. How do you make the decision to stop feeding your children (portion sizes)?

7. Have you faced any problems with feed your child. If you managed to overcome this problem, how?

Acculturation

8. Is there different culture between Chinese and British food culture and How do you think the food culture is different between the British and Chinese?

9. Do you give any traditional remedies to a child if he or she is feeling poorly?

10. What culture factors affect your health ideas and practices? Which do you feel are most important?

11. At present, most of time what factors affect you choose foods?
a. Chinese or British foods?
b. Chinese or British cooking methods?

12. If you think there is feeding culture conflict between British culture and Chinese culture, what factors influence your choice?

13. How do the different food cultures influence the way in which you feed your children?
